# Crystal structure of an antigenic outer-membrane protein from *Salmonella* Typhi suggests a potential antigenic loop and an efflux mechanism

**DOI:** 10.1038/srep16441

**Published:** 2015-11-13

**Authors:** Hong-Hsiang Guan, Masato Yoshimura, Phimonphan Chuankhayan, Chien-Chih Lin, Nai-Chi Chen, Ming-Chi Yang, Asma Ismail, Hoong-Kun Fun, Chun-Jung Chen

**Affiliations:** 1 Life Science Group, Scientific Research Division, National Synchrotron Radiation Research Center, Hsinchu, 30076, Taiwan; 2 Institute of Biotechnology, and University Center for Bioscience and Biotechnology, National Cheng Kung University, Tainan City, 701, Taiwan; 3 Institute for Research in Molecular Medicine, Universiti Sains Malaysia, Kelantan, Malaysia; 4 Department of Pharmaceutical Chemistry, College of Pharmacy, King Saud University, Riyadh, 11451, Saudi Arabia; 5 X-ray Crystallography Unit, School of Physics, Universiti Sains Malaysia, 11800 USM, Penang, Malaysia; 6 Department of Physics, National Tsing Hua University, Hsinchu, 30043, Taiwan

## Abstract

ST50, an outer-membrane component of the multi-drug efflux system from *Salmonella enterica* serovar Typhi, is an obligatory diagnostic antigen for typhoid fever. ST50 is an excellent and unique diagnostic antigen with 95% specificity and 90% sensitivity and is used in the commercial diagnosis test kit (TYPHIDOT^TM^). The crystal structure of ST50 at a resolution of 2.98 Å reveals a trimer that forms an α-helical tunnel and a β-barrel transmembrane channel traversing the periplasmic space and outer membrane. Structural investigations suggest significant conformational variations in the extracellular loop regions, especially extracellular loop 2. This is the location of the most plausible antibody-binding domain that could be used to target the design of new antigenic epitopes for the development of better diagnostics or drugs for the treatment of typhoid fever. A molecule of the detergent n-octyl-β-D-glucoside is observed in the D-cage, which comprises three sets of Asp361 and Asp371 residues at the periplasmic entrance. These structural insights suggest a possible substrate transport mechanism in which the substrate first binds at the periplasmic entrance of ST50 and subsequently, via iris-like structural movements to open the periplasmic end, penetrates the periplasmic domain for efflux pumping of molecules, including poisonous metabolites or xenobiotics, for excretion outside the pathogen.

*Salmonella*, a genus of rod-shaped, Gram-negative bacteria, comprises two species, *Salmonella enterica* (*S*. *enterica*) and *Salmonella bongori* (*S. bongori*). *S. enterica* is subdivided into more than 2,000 serovars, of which *Salmonella* Typhi (*S.* Typhi) infects only humans and is a leading agent for typhoid fever, also known as enteric fever, a potentially fatal disease that causes multi-systemic illness^1^. In total, approximately 21 million new cases of typhoid fever and over 200,000 deaths annually occur worldwide. Even in a developed country, such as the USA, approximately 6,000 new cases of typhoid fever occur each year. Currently, the principal method to diagnose typhoid fever is TYPHIDOT, a rapid immunoassay test to detect IgM and IgG antibodies against the outer-membrane protein ST50 of *S.* Typhi^2^,^3^.

The gene (1476 bp, accession no. BD079162) encoding ST50, also known as the outer-membrane protein TolC precursor in *S.* Typhi (accession no. AB0890), has been used together with the basic local alignment search tool (BLAST) to search for sequence similarities against the GenBank. The results show that this gene exists in both the genomes of *Salmonella enterica* subsp. *enterica* serovar Typhi strain Ty2 (accession no. AAO70650) and CT18 (accession no. CAD07712). The Typhi strain Ty2 is the human-specific pathogen causing typhoid fever, and its gene sequence has been the foundation for vaccine development^4^. CT18 carries an additional multi-drug-resistant plasmid compared to the strain Ty2^5^.

ST50, an outer-membrane protein with the molecular mass of approximately 50 kDa, is expected to be a tripartite member of the resistance-nodulation-cell division (RND) efflux system because of its high sequence identity with TolC (89%), one component of the drug efflux system AcrA-AcrB-TolC in *E. coli*^6^,^7^. Before our work, three crystal structures of outer-membrane components of these multidrug efflux pumps, TolC, Oprm and VceC,^8^,^9^,^10^,^11^ have been determined. In addition, two electron microscopy structures of the entire AcrA-AcrB-TolC multidrug efflux pumps with varying assembly modes have been reported^12^,^13^. One outer-membrane component of the heavy metal efflux pump, CusC, also possesses an architecture similar to those of the outer-membrane multidrug efflux pumps^14^. These tripartite efflux pumps are able to transport diverse substrates, including metal ions, dyes, detergents, antibiotics and protein toxins, from the cytoplasm to the external medium of the microorganism^15^. During transport of these substrates, two putative gates, the extracellular loops and the periplasmic entrance, located at the two ends of these outer-membrane proteins play important roles in passing substrates across the outer membrane. However, the mechanisms of opening and closing of the two gates, including how the switching is controlled, are not fully understood.

One iris-like mechanism has been proposed, which suggests opening of the periplasmic entrance via helix twisting and breaking both intra-monomeric and inter-monomeric hydrogen bonds and electrostatic interactions near the entrance^16^. However, the relationship between the phenomenon of opening and closing the extracellular loops and the gating mechanism of the periplasmic entrance is not well understood. Simulations by molecular dynamics (MD) have elucidated the conformational dynamics of the extracellular loops that might be related to β-channel gating and the consequent closure of the extracellular mouth in the TolC structure^17^. However, simulations of TolC in a phospholipid membrane/150 mM NaCl solution environment did not reveal a gating mechanism on the extracellular side^18^. Thus, the issue of a gate on the outer membrane remains to be addressed.

To identify the immunogenic site of ST50 and investigate the structural features of this antigenic outer-membrane protein, we overexpressed ST50 from *Salmonella enterica* serovar Typhi in *E. coli* and determined its structure at a resolution of 2.98 Å. A detailed structural comparison of ST50 with other homologues allowed us to delineate the immuno-dominant site of ST50 and elucidate the mechanism of substrate efflux.

## Results

### Protein purification and characterization

During protein purification in the early stage by size-exclusion chromatography, a buffer containing 0.04% β-DDM was utilized to elute the ST50 protein. Most ST50, however, ended up in the aggregation fraction (V_o_) during the elution. To solve this problem, the amount of β-DDM was increased to a final concentration 0.2% for size-exclusion chromatography. The size-exclusion profile with β-DDM (0.2%) showed a major fraction with a molecular mass of approximately 220 kDa, in agreement with an expected total molecular mass of the trimeric ST50 (150 kDa) encapsulated in a detergent micelle containing 137 molecules of β-DDM (approximately 70 kDa) (Supplementary Fig. S1a). SDS-PAGE showed that purified ST50 had greater than 90% purity (Supplementary Fig. S1b). Analyses of ST50 by both conventional circular dichroism (CD) and synchrotron-radiation CD (SRCD) spectra showed that β-DDM-solubilized ST50 is an α-helical-dominant protein in solution. A comparison of the secondary structure ratios, which were calculated from the SRCD data (helix 58.5%; sheet 7.2%) (Supplementary Fig. S2), predicted from software tools (averaged helix 60.8%; sheet 7.7%) (Supplementary Table S1), and estimated from the structure of ST50 determined in this study (*vide infra*) (helix 61%; sheet 13%), shows reasonable agreement in the amount of helices but not β-strand structures, given the large conformational flexibility between β-strands in the transmembrane domains and non-β-strand structures in the extracellular loops.

### Overall structural architecture

**Figure 1:**
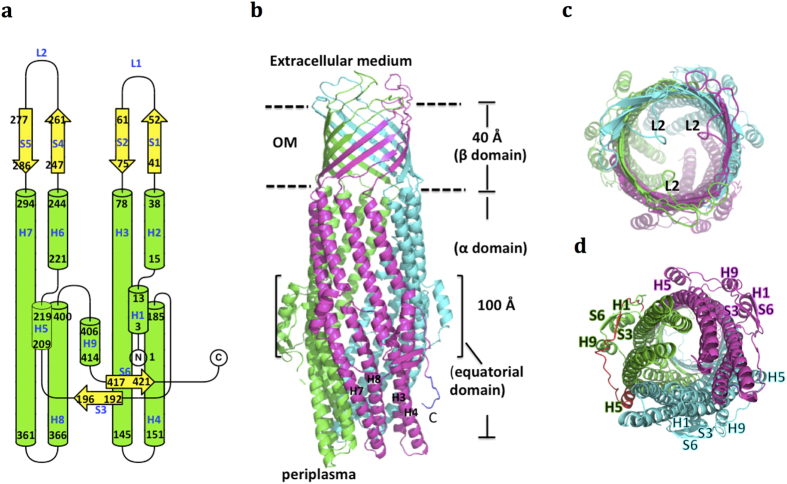
Overall architecture of trimeric ST50. (**a**) The topology diagram of one ST50 protomer. The figure was prepared using *TopDraw* in the *CCP4* suite. Helices and sheets are labeled with green and yellow, respectively. (**b**) The cartoon view is perpendicular to the trimeric three-fold axis. In the α-helical domain, the inner coiled-coil, H3/H4, and the outer coiled-coil, H7/H8, are labeled in black. C represents the C-terminus of the C protomer ST50, and the additional 8 residues built on ST50, compared to TolC, are labeled in blue. (**c**) A view of ST50 from the extracellular medium. L2 denotes extracellular loop 2 (a.a. 262 to 276) between S4 and S5. (**d**) A view of ST50 from the cytoplasmic membrane. The secondary structures in the equatorial domain are labeled. The residues 187 to 220 of the protomer A are labeled in red.

ST50 exhibits a typical TolC-like structural repeat in one protomer (Fig. 1a). In one asymmetric unit, three protomers of ST50 form a functional trimer through interactions of the interprotomer H7/(adjacent H2, H4) coiled-coil and the antiparallel β-sheet S5/(adjacent S1) (Supplementary Fig. S3). The non-crystallographic three-fold symmetry allows three protomers of ST50 to exhibit crucial structural differences. ST50, similar to other outer-membrane proteins involved in resistance-nodulation-cell division (RND) efflux systems, possesses an α-helical barrel domain, a membrane-embedded β-barrel domain and an equatorial domain of mixed α/β-structures (Fig. 1b). The α-helical barrel domain, with a tunnel of length 100 Å across the periplasmic space, comprises 12 long helices of which H7/H8 form the α-helical inner coiled-coil and H3/H4 form the outer coiled-coil in each protomer. The β-barrel domain, a transmembrane channel with a length of approximately 40 Å, consists of 12 β-strands of which S1-S2 form the antiparallel β-sheet with a short extracellular loop (L1), and S4–S5 form the antiparallel β-sheet with a long extracellular loop (L2) in each protomer (Fig. 1a,c). The equatorial domain surrounding the middle of the periplasmic tunnel contains the N-terminal region from residues 1 to 14 and residues 187 to 220 (Fig. 1d), including S3 (residues 193–196) and H5 (residues 209–219), and the C-terminal region from residues 402 to 425 in protomers A and B and from residues 402 to 433 in protomer C. The N-terminal region contains H1 (residues 3–13), and the C-terminal region contains H9 (residues 406–414) and S6 (residues 417–421) in each protomer.

The long extracellular loops L2 between β-strands S4 and S5 exhibit varied conformations among the three protomers, and the B-chain possesses the largest conformational deviation (Supplementary Fig. S4). In addition, different from the structure of truncated TolC without the last 43 residues at the C-terminus, ST50 contains the complete sequence with an intact C-terminal region. Based on the interpretable electron density, the additional 8 residues (residues 426–433) of the C-chain structure could be built on to the structure of ST50 (Fig. 1b), in comparison with the truncated TolC. In TolC, the lower half of the α-helical domain was suggested to interact with its tripartite partner, AcrAB^19^,^20^. The fragment of the additional observed 8 residues on the C-terminal end of ST50, also located at the lower half of the α-helical domain, extends to the periplasmic entrance (Fig. 1b). The C-terminus of ST50 may be involved in binding to its cognate tripartite partners because large sequence variations in the C-terminal regions among the outer-membrane proteins (OMPs) exists (Supplementary Fig. S5).

### Structural comparison of ST50 and other OMPs

The root-mean-square-deviation (RMSD) between the overall structures of ST50 and TolC, Oprm and VceC are 0.854, 2.266 and 1.858 Å, respectively, which were calculated with the secondary structure matching method using the *superpose* program in the *CCP4* suite. The periplasmic entrance of ST50 adopts the resting closed state through close interactions between the α-helical inner coiled-coil (H7/H8) and the outer coiled-coil (H3/H4) through hydrogen bond networks^21^, similar to those of TolC, Oprm and VceC^8^,^9^,^10^. The hydrogen bond network comprises four key residues: Thr152, Asp153, Tyr359 and Arg364 in ST50, corresponding to Thr152, Asp153, Tyr362 and Arg367 in TolC. The intraprotomer hydrogen bond Tyr359–Asp153 links the inner coiled-coil (H7/H8) and outer coiled-coil (H3/H4). Arg364, located at the small loop between H7 and H8, forms interprotomer hydrogen bonds (Arg364–Thr152 and Arg364–Asp153) with Thr152 and Asp153 on H4 of the adjacent protomer (Supplementary Fig. S6). This pattern of the hydrogen bond network in ST50 is similar to that in TolC. Disruption of the analogous hydrogen bonds in TolC opens the periplasmic entrance^16^,^22^.

**Figure 2:**
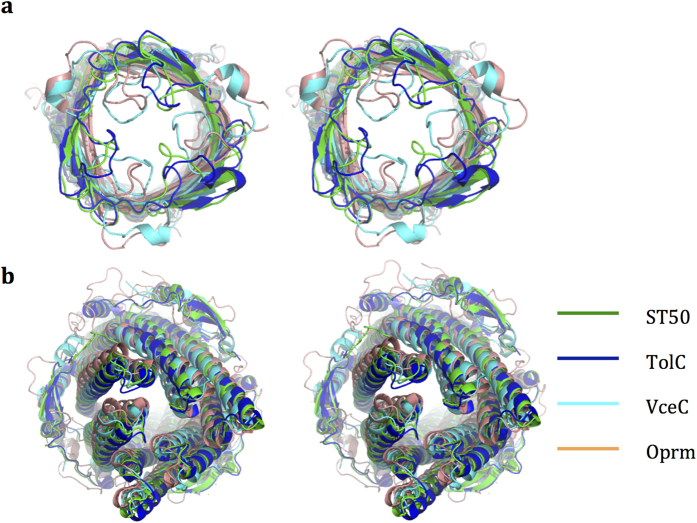
Stereo views of superimposed TolC-like structures, ST50, TolC, VceC and Oprm. (**a**) A view from the extracellular medium. (**b**) A view from the cytoplasmic membrane. ST50, TolC, VceC and Oprm are colored green, blue, cyan, and brown, respectively.

The β-barrel of ST50 forms a cylinder-like structure. Comparably, the β-barrels of TolC and VceC are both cylindrical, whereas that of Oprm resembles a triangular prism. The triangular prism is proposed as a more stable conformation of the β-barrel, and TolC may adopt the triangular-prism conformation when it is released from the crystallographic environment^17^. As shown in Fig. 2a,b, the RMSD in β-barrel and extracellular-loop domains between the structures of ST50 and TolC, Oprm and VceC are 0.874, 2.712 and 2.250 Å, respectively, whereas the RMSD in α-barrel and equatorial domains between the structures of ST50 and TolC, Oprm and VceC are 0.616, 2.634 and 1.906 Å, respectively. The substantial conformational variations among the structures of TolC, Oprm, VceC and ST50 are mainly located at the equatorial domains and extracellular loops. The equatorial domain variations among these OMPs might be utilized to accommodate the individual tripartite partners because the equatorial domains are implicated in tripartite partner binding to perform drug efflux functions according to previous work^21^. The extracellular loops in the structure are other regions with varied conformations among the four protein structures being compared (Fig. 2a); coincidentally, they are also the most dynamic regions in ST50. The great flexibility of the extracellular loops was also found in several OMPs and is expected to be involved in the mechanism of outer-membrane channel gating^23^,^24^.

### The extracellular loop L2 occludes the β-barrel channel entrance

**Figure 3:**
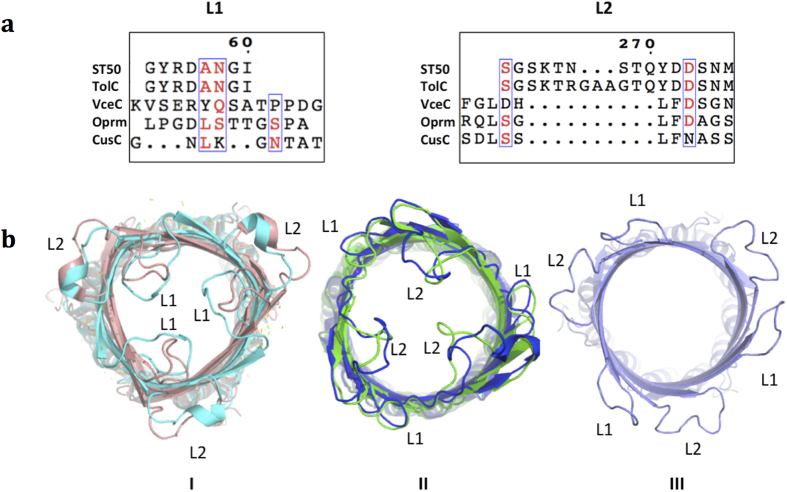
Extracellular loops of three types of the resistance-nodulation-division (RND) pumps. (**a**) Sequence alignments of extracellular loop 1 (L1) and extracellular loop 2 (L2) for five proteins of the RND family. (**b**) VceC (cyan) and Oprm (brown) are classified as type I. ST50 (green) and TolC (blue) are classified as type II. CusC (purple) is classified as type III.

A structural comparison of tripartite pumps of OMPs allows classification of the extracellular loops into three distinct types (Fig. 3b): (**I**) The L1 type, found in Oprm and VceC, possesses a longer L1 loop with four and six residues more than those of TolC and ST50, respectively, occluding the β-barrel channel by resting over the channel entrance. (**II**) The L2 type, found in ST50 and TolC, possesses a longer L2 loop, with four and seven residues more than those of Oprm and VceC, respectively, occluding the β-barrel channel by resting over the upper side of the channel entrance. The proteins of type I adopt a conformation of the β-barrel channel that is more open than that in the proteins of type II. (**III**) The open type, such as CusC, adopts a completely open form of the β-barrel channel with shorter L1 and L2 loops^14^ (Fig. 3a,b). The multidrug efflux OMP seems to utilize only one of the two extracellular loops, the longer loop, to occlude the transmembrane channel. The space of the channel entrance is inadequate to accommodate two long loops together. The long L2 loop might thus be critical for substrate efflux in ST50.

### π-stacking interactions in the ST50 extracellular loop

**Figure 4:**
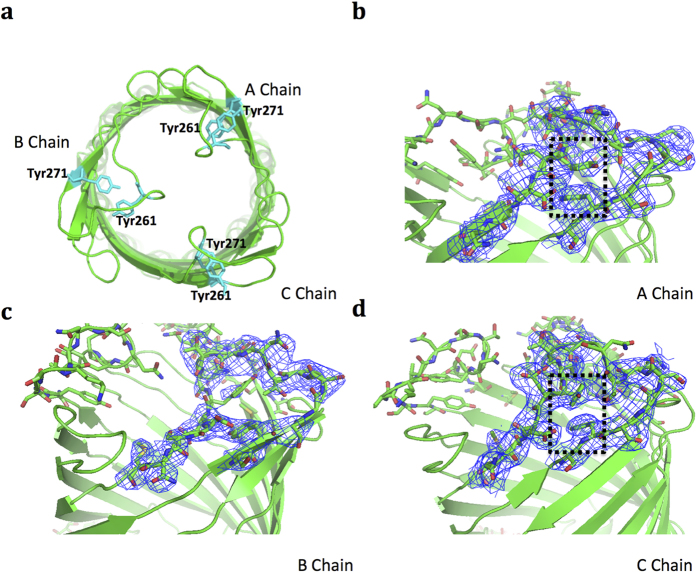
The π-stacking interaction in extracellular loop 2 (L2) of ST50. (**a**) The π-stacking residues are shown (in cyan sticks) in extracellular loop 2. Tyr261 and Tyr271 form π-stacking interactions in protomers A and C but not in the protomer B. The 2*F*_o_*-F*_c_ electron density maps of the extracellular loop 2 (L2) of chain A (**b**), chain B (**c**) and chain C (**d**) are shown (blue meshes). The π-stacking interactions between Tyr261 and Tyr271 are indicated with dashed-line rectangles.

ST50 utilizes the longer extracellular L2 loop to act as a transmembrane channel cap, as in the case of TolC. The L2 loop (a.a. 262 to 276) of ST50 is three residues less than that of TolC. π-stacking interactions between Tyr261 and Tyr271 are found in the A and C protomers but not in the B protomer of ST50 (Fig. 4). The distinct conformation and the large temperature factor of the L2 loop in the B protomer (Supplementary Fig. S7) result from compression on the L2 loop by helix 1 of another adjacent ST50 molecule that is related to crystal packing (*θ/*A-protomer = 103.1°, *θ/*C-protomer = 100.2°, *θ/*B-protomer = 71.8°). The dihedral *θ* angles, with similar definition to that in TolC^18^, were calculated from the Cα atoms of three Asp56 in the same plane on the β-barrel and Ser268 at the tip of each extracellular loop based on the same criteria of TolC for comparison. The larger *θ* angle might represent a more open extracellular loop. Previous MD simulations on the extracellular loops of TolC showed all possible loop conformations with *θ* ranging from -30 to 180° with two major maxima at 30° and 135°^18^, compared to only limited possibilities of the loop conformations, with the *θ* range of 87.1 to 111.5*°* observed in all X-ray structures of known wild-type and mutant TolC. The extracellular loop of ST50 also adopts a partially open conformation, as observed in TolC, and possesses a *θ* angle similar to TolC, suggesting that the *θ* range of 87.1 to 111.5*°* observed in all TolC structures and the ST50 structure might represent one resting state of minimum energy. In addition, the π-stacking interactions between Tyr261 and Tyr271 found in the ST50 structure ensures that the conformation of the loop is in a preferred state. When ST50 is effluxing substrates to the extracellular environment, the π-stacking interactions might be altered to switch the loop to an open conformation for this efflux. In contrast, when ST50 is not effluxing substrates, it might maintain a partially open conformation (*θ* range from 87.1 to 111.5*°*) via π-stacking interactions to prevent outside small substrates from passing into the cell through the membrane channel.

### Bound detergents in ST50

**Figure 5:**
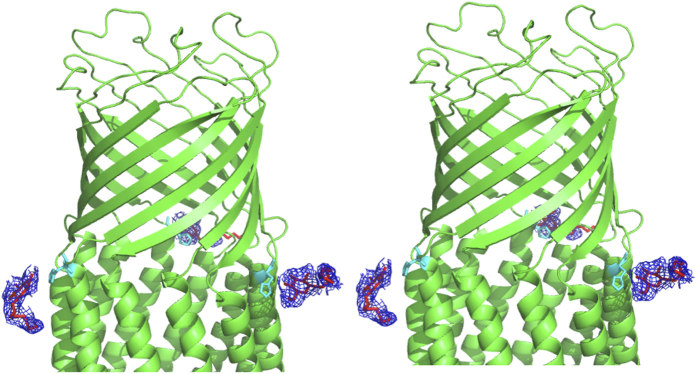
A stereo view of bound detergents n-octyl-β-D-glucoside (β-OG) in ST50. The bound β-OG molecules (in red sticks) interact with His244 residues (cyan sticks) via hydrogen bonds. The 2*F*_o_*-F*_c_ composite simulated omit electron density map of detergents is shown (blue meshes).

Four bound detergent molecules of n-octyl-β-D-glucoside (β-OG) are found in the structure of ST50. His244 of all three ST50 protomers interact with the β-OG molecules through hydrogen bonding between the imidazole ring of His244 and the polar group of β-OG (Fig. 5). His244 is located at an intersection between one β-strand of the β-barrel and one α-helix of the α-helical barrel within the same protomer. From a view of the functional trimer of ST50 that forms the channel-tunnel architecture, these His244 residues are located at the bottom of the transmembrane channel; the bound β-OG molecules are considered mimics of membrane lipids, suggesting that the transmembrane region of ST50 might utilize His244 to interact with the polar head groups in the inner leaflet of the lipid layer of the outer membrane.

**Figure 6:**
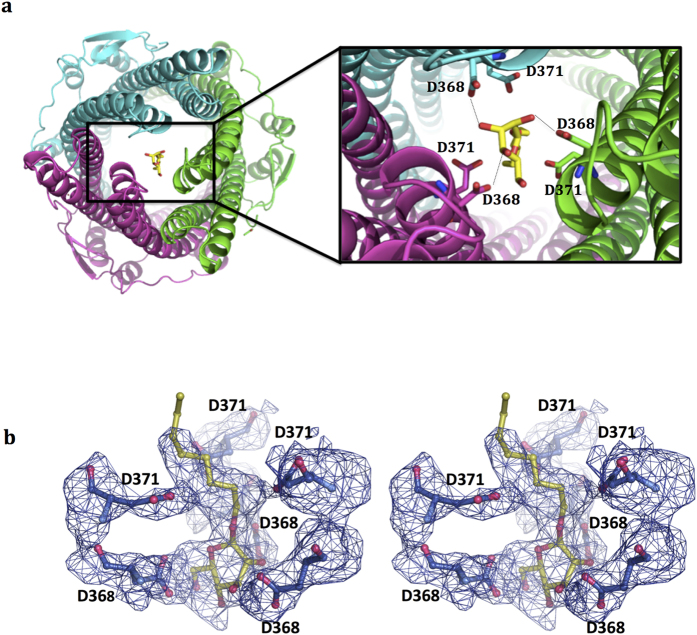
Bound β-OG at the periplasmic entrance. (**a**) The cartoon view perpendicular to the membrane (the left figure) shows that the detergent β–OG blocks the periplasmic entrance of ST50. The hydrogen bonds between the polar head of β–OG and D368 of ST50 are labeled with black dashes. (**b**) A stereo view of the D-cage (dark blue sticks) and the bound detergent β–OG (yellow stick). The 2*F*_o_*−F*_c_ composite simulated omit electron density map, generated by the *PHENIX* suite, is shown as blue meshes at the contour level of 1.0 σ.

Another β-OG molecule is identified at the entrance of the periplasmic tunnel, where hexamminecobalt, the potent blocker of TolC and ST50, has also been located^2^,^25^. Apart from hexamminecobalt, two ions, Na^+^ and K^+^, were also reported to bind to TolC near the periplasmic entrance based on computer simulations. Only the Na^+^ is located at a position similar to the observed β-OG^17^,^25^. The polar head group of β-OG forms hydrogen bonds with Asp368 (structural equivalent to Asp371 in TolC) (Fig. 6a), rather than Asp371 (equivalent to Asp374 in TolC that forms hydrogen bonds mainly with hexamminecobalt). The hydrophobic tail of this β-OG is, however, not clearly visible because of poor electron density in this region (Fig. 6b). The three sets of Asp368 and Asp371 residues in ST50 (equivalent to Asp371 and Asp374 in TolC) seem to be able to form a cage-like geometry to trap small molecules through electrostatic interactions or hydrogen bond forces at the periplasmic entrance while these outer-membrane proteins are in the closed resting state. We define this newly identified structural geometry comprising six Asp residues as the Asp-cage or D-cage.

## Discussion

Conservation of the amino acid sequence of extracellular loops among ST50, TolC, Oprm and VceC is much lower than that of other regions, except for the equatorial domain. With the large structural variations and extracellular accessibilities, the extracellular loops, short L1 (a.a. 53–60) and long L2 (a.a. 262–276) of ST50, are the most likely recognition sites by the antibody in patients diagnosed with typhoid fever. The two loop regions are mainly exposed to the extracellular medium, a target that can elicit an antibody from the host. However, both L1s in ST50 and TolC share the same primary sequence (Fig. 3a and Supplementary Fig. S5) and adopt similar structural conformations (Fig. 2b), indicating that the L2 loop might have the specificity to play an important role in antibody recognition and be the immuno-dominant site in ST50. In addition, according to ELISA experiments in previous work^27^, denatured ST50 possesses reactivity against sera of typhoid patients, indicating that the discontinuous amino acid sequences of ST50 are less crucial for antibody binding, whereas the linear amino acid sequence of ST50 might serve as an epitope for paratope binding. These analyses suggest that the linear amino acid sequence of L2 in ST50 might be a primary candidate for the development of an optimized diagnostic antigen or a new drug target for typhoid fever.

Our structure reveals that one β-OG molecule is bound at the D-cage, which comprises three sets of Asp368 and Asp371 residues, at the entrance of the periplasmic tunnel. With the OMP in the closed resting state, the small substrate molecules are trapped at the D-cage near the periplasmic entrance through electrostatic or hydrogen bond interactions. For example, Asp371 and Asp374, which form the inner bottleneck (BNI), interact with Na^+^ in a proposed sodium-dependent lock mechanism, based on a previous simulation on TolC^18^. A D-cage of this type might explain why the D371A mutation of TolC also has an unexpectedly severe effect on ligand binding, although no significant interactions involving Asp371 were observed in the structure of the TolC-hexamminecobalt complex^25^. It can also explain why the single mutations D368A and D371A and a double mutation D368A/D371A in ST50 greatly decrease the binding affinity toward hexamminecobalt^2^. This D-cage might serve as a controller to maintain substrates in a starting line before efflux. Subsequently, to initiate efflux, the hydrogen bond networks created by the Thr152, Asp153, Tyr359 and Arg364 residues are broken, opening the periplasmic entrance to begin substrate passage into the outer-membrane channel of ST50.

The gating mechanism of ST50 between the external and internal gates on the extracellular side and the periplasmic entrance of the protein remains unclear. ST50 was crystallized at an acidic pH, whereas the homologue TolC was crystallized in neutral or basic conditions. Despite the different crystallization conditions, the extracellular loops, especially the L2 loops, of ST50 and TolC all adopt similar structural conformations, indicating that the switching mechanism of extracellular loops in ST50 and TolC might not depend on pH. In contrast, the gating mechanism in OmpG is pH-dependent^24^. We have monitored the dihedral *θ* angle*s* calculated from the Cα atoms of three Asp56 in the same plane of the β-barrel and Ser268 in each extracellular loop. The extracellular loops of ST50 adopt a *θ* angle similar to that of TolC (PDB entry 1EK9, average *θ* = 89.8°) with a partially open state (ST50, acceptable efflux compounds with diameters <5–6 Å; TolC, acceptable efflux compounds with diameters <7–8 Å)^8^,^18^,^28^ (Fig. 3b), indicating that extracellular loop conformations in ST50 and TolC might be in a resting state in the extracellular region. With the efflux of large substrates, the conformation of the extracellular loops must be altered, in contrast to previous simulations that suggested no gating behavior at the extracellular side of TolC^18^. This gating mechanism paradox at the extracellular side of ST50 requires further studies.

The crystal structure of ST50 reveals partially open extracellular loops (L2), with a stacking interaction between Tyr261 and Tyr271 and a detergent β-OG bound in the D-cage at the resting closed periplasmic entrance. The partially open conformation of the L2 loops might be a preferred conformation of TolC and ST50. All L2 loops in the X-ray structures of ST50 and TolC-related proteins exhibit similar conformations with *θ* range of 87.1 to 111.5*°*, suggesting that the observed conformations of L2 are specific for the closed resting state of ST50 and TolC, rather than the arbitrary snapshot structures in crystals. Presumably, the stacking interactions between Tyr residues found in ST50 and all TolC-related structures contribute to stabilizing this preferred conformation.

**Figure 7:**
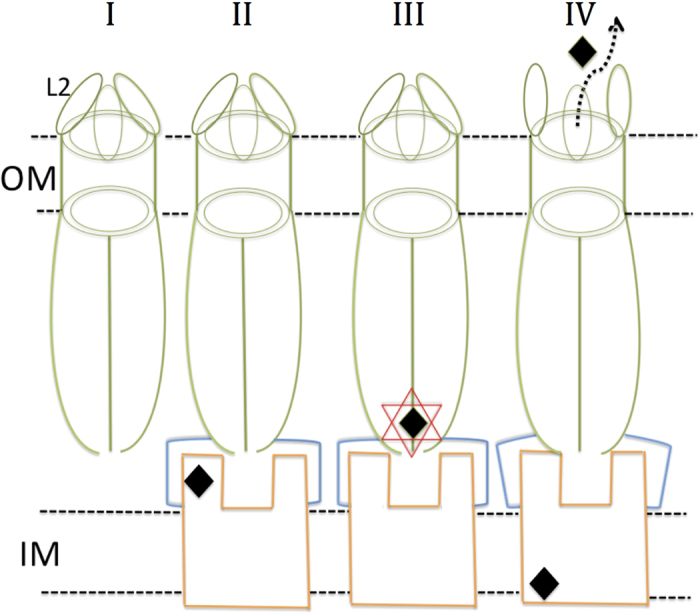
Four stages of substrate efflux of the ST50 pump. Stage **I**. In the absence of the efflux substrate, the extracellular loop (L2) adopts a partially open conformation, and the periplasmic entrance presents a resting closed state as the observed periplasmic entrance conformations of ST50 and wild-type TolC. Stage **II**. The efflux substrate binds to the compatible inner-membrane transporter of ST50, and ST50 connects to its membrane fusion protein (MFP) in the periplasmic space as well as the compatible inner-membrane protein (IMP) to form a functional efflux pump. Stage **III**. The efflux substrate flows and binds into the D-cage in the periplasmic entrance of ST50. Stage **IV**. The periplasmic entrance of ST50 transits from the resting closed state to the open state, and the substrate passes the outer membrane via ST50 while the gate formed by three extracellular loops (L2) is open. ST50 and the cognate proteins MFP and IMP are colored green, blue and orange, respectively. The black diamonds denote small efflux substrates and the red star represents the D-cage, comprising three sets of Asp368 and Asp371 residues on three protomers.

Based on the structure of ST50 determined in this study, a comparison of related structures, and the proposed iris-like gating mechanism at the periplasmic entrance^8^, we suggest a mechanism of substrate efflux (Fig. 7). In **stage I** of the model, the partially open conformation (*θ* range from 87.1 to 111.5°) of L2 limits substrates in the extracellular environment to flow into the periplasmic space; the periplasmic entrance is in the closed resting state. In stage **II**, the efflux substrate binds to the compatible inner-membrane transporter of ST50; ST50 interacts with its membrane fusion protein (MFP) in the periplasmic space and the compatible inner-membrane protein (IMP) to form a tripartite functional efflux pump. In stage **III**, the substrate can be accepted at the D-cage in the periplasmic entrance of ST50 from its inner-membrane cognate partner even when ST50 adopts a closed resting conformation, as a small detergent molecule β-OG was observed at the D-cage in our structure. There is another possibility that the transfer and receipt of substrates from other single-component efflux transporters occurs on the inner membrane, such as a major facilitator and small-molecule-resistance superfamilies rather than compatible tripartite IMP^29^. The observation of a detergent substrate in the D-cage of the close periplasmic entrance might support these two possible processes.

The previously reported structure of AcrB, a compatible inner-membrane transporter of TolC, showed that three protomers of AcrB adopt asymmetric conformations, among which only one protomer binds the substrate that was exported according to a rotating mechanism^30^,^31^,^32^. No significant conformational modification was observed in the TolC-docking domain of AcrB during the binding change cycle^30^, suggesting that the opening of the TolC periplasmic entrance by an iris-like mechanism might result from a rearrangement of MFP, AcrA, rather than binding with IMP, AcrB. The iris-like mechanism might arise from the rearrangement of MFP via a proposed interaction cogwheel-to-cogwheel between MFP and OMP^13^,^33^. In stage **IV**, ST50 begins efflux movement. The periplasmic entrance is opened with an iris-like mechanism upon breaking the hydrogen bond network near the periplasmic entrance; the substrate is subsequently released from the D-cage. The iris-like movement of the periplasmic entrance might be triggered by the cognate MFP of ST50; the substrate subsequently moves through the outer-membrane channel via the open extracellular loops into the extracellular space.

The gating mechanism with the extracellular loops (L2) is still puzzling. It is also plausible that a gate formed by extracellular loops is not opened by the channel itself but is pushed out and opened by moving efflux substrates. This behavior might also explain why the extracellular loops exhibit flexible structures rather than rigid ones. In addition, in the B protomer of ST50, the parallel-displaced stacking interaction is disrupted; the conformation of L2 is altered and different from those of A and C protomers by extrusion from an adjacent ST50 molecule in the crystal, indicating that the gate could be opened from the partially open state by the pushing movement of substrates, as shown in stage **IV**.

In summary, this X-ray crystallographic study of ST50 of *S.* Typhi provides data regarding the plausible immuno-dominant site of this important antigenic protein. We suggest that the most plausible antigenic site is located at the extracellular loop L2, based on an extensive structural comparison among ST50 and other OMPs. In addition, L2 adopts a partially open conformation, similar to the TolC structure, with π-stacking interactions between Tyr261 and Tyr271, indicating that a gating mechanism on the extracellular end is required to alter the specific conformation to efflux substrates. Together with a bound detergent observed in the D-cage at the closed resting periplasmic entrance, formed by the Asp368 and Asp371 residues from each of the three protomers, we propose a novel small-molecule efflux mechanism for the tripartite efflux machine.

## Methods

### Protein expression and purification

To obtain a large amount of ST50 protein (a.a. 25 to 491), the gene encoding ST50 without the signal peptide, *st50 (*Δ*24*), was cloned into the PHDST plasmid vector. The PHDST-ST50 plasmid was then transformed into BL21(DE3) cells. The cells carrying the PHDST-ST50 plasmid were cultured in 3 mL LB with ampicillin (120 μg/mL) at 37 °C with vigorous shaking overnight. The 2-mL expression culture was incubated with LB (1 L) at a 1:500 dilution with ampicillin (120 μg/mL). The expression cells (1 L) were cultivated at 37 °C; the optical density (OD) of the culture was measured and monitored by a wavelength 595 nm with a spectrometer (Varian). After OD_595_ of the cultures reached 0.4–0.6, isopropyl-β-D-1-thiogalactopyranoside (IPTG) was added (final concentration 1 mM) to induce the culture for overexpression of ST50. The overexpressed cells were harvested by centrifugation at 10,000 × *g* for 25 min at 4 °C. The cell pellet was dissolved in a buffer (20 mL) containing Tris (20 mM, pH 7.5) and n-dodecyl-β-D-maltoside (β-DDM, 2%) and then sonicated with a probe (diameter 1 cm) for 10 min at a temperature below 10 °C. The sonicated suspension was centrifuged at 10,000 × *g* for 25 min at 4 °C; the supernatant was collected and applied to a Ni-NTA chromatography column with the wash buffer (20 mM Tris, pH 7.5, 0.2% β-DDM, 100 mM imidazole) followed by the elution buffer (20 mM Tris, pH 7.5, 0.2% β-DDM, 200 mM imidazole). The elution fraction was collected and dialyzed with buffer (Tris 20 mM, pH = 7.5, β-DDM 0.2%) overnight. Sumo protease was added to the dialyzed protein solution to remove the sumo tag. Ni-NTA chromatography was used again to remove uncut ST50 and sumo protease. The flow-through fraction containing ST50 without the sumo tag was subsequently applied to an anion-exchange column (Hitrap QFF). A size-exclusion column (Superdex 200 10/30 GL) was utilized with buffer (Tris 20 mM, pH 7.5, NaCl 150 mM, β-DDM 0.2%) to suppress the aggregation of ST50. ST50 in its pure trimeric form was then collected.

### Secondary structure analyses

To eliminate the strong chloride effect in the far UV region, especially below 200 nm in the circular dichroism spectra recorded with synchrotron radiation (SRCD), the ST50 buffer solution was changed to a double-distilled-H_2_O buffer containing only DDM (0.2%). Because an accurate determination of the sample concentration is critical for analysis of SRCD spectra, the measurement of the concentration of ST50 was repeated more than three times (using NanoVue, GE Healthcare). Conventional CD measurements were performed using a circular dichroism spectrometer (AVIV Model 410), whereas SRCD spectra were measured at beamline 04 of NSRRC (Hsinchu, Taiwan). Analyses of both conventional CD spectra and SRCD spectra were performed with algorithms *K2D* and *SELCON3*, respectively, in the *DichroWeb* server^34^. The predicted secondary structure content was calculated with prediction tools *SAM*, *PSIPRED*, *PredictProtein*, *SABLE* and *YAPSIN* based on the primary sequence of ST50.

### Crystallization and X-ray data collection

Purified ST50 was concentrated to 8 mg/mL using a centrifugal filter (Amicon Ultra-15) with the molecular mass cut-off at 100 kDa. Initial crystal screenings were performed using a robot (Mosquito Crystal, TTP Labtech) with the hanging-drop vapor-diffusion method on mixing the protein sample (100 nL) with a reservoir solution (100 nL) from the screen kits. Small crystals of sizes 0.1 × 0.04 × 0.04 mm^3^ were observed in one condition with sodium acetate (100 mM, pH 4.6), NaCl (200 mM), PEG 8000 (15%) at 18 °C after two weeks. Crystals of ST50 were first transferred to a crystallization buffer containing PEG 400 (15%) for 30 s for cryoprotection and were immediately frozen in liquid nitrogen. The X-ray diffraction experiments were performed on beamlines of BL15A1 at the NSRRC (National Synchrotron Radiation Research Center, Hsinchu, Taiwan) and BL44XU and BL12B2 at SPring-8 (Hyogo, Japan) with a wavelength 0.9 Å. These screened crystals initially diffracted to only approximately 20 Å with few diffraction spots but showed a promising diffraction pattern of protein crystals. Protein crystals were further optimized using hanging drops consisting of equal volumes of protein solution (1 μL) and reservoir solution (1 μL) equilibrated against 150 mL reservoir solution consisting of sodium acetate (100 mM, pH 4.6), NaCl (86 mM), PEG 8000 (10.4%) and β-OG (0.4%) at 18 °C after two weeks. The improved larger crystals grew to a final size of 0.3 × 0.1 × 0.1 mm. However, these crystals diffracted to only approximately 7.5 Å. Dehydration of protein crystals was subsequently utilized^35^, and finally the best complete data set at a resolution 2.98 Å was collected at BL44XU after more than one hundred crystals were screened simultaneously at BL12B2 of SPring-8. The data were processed with *HKL2000*. All X-ray data statistics are summarized in Table 1.

### Structure determination

The processed data were reduced from averaged intensities to mean amplitudes with *TRUNCATE* in *CCP4* suite^36^. The initial phase was determined by the molecular replacement method using *MOLREP*^37^ in *CCP4* suite with a polyalanine backbone of TolC (PDB entry 1ek9) as the search model. Iterative cycles of model building and refinement using *COOT*^38^ and *REFMAC*^39^, respectively, were performed. In particular, the largest conformational variations between the electron density map and the initial model were observed at the extracellular loop (L2) between β-strands 4 and 5. The fragment of L2 from residues 262 to 275 in the search model was removed and re-built based on the 2*F*_o_*–F*_c_, *F*_o_*–F*_c_ and composite simulated omit electron density maps. Detergent molecules, n-octyl-β-D-glucoside, were fitted into appropriate positive *F*_o_*–F*_c_ electron density maps. Iterative cycles of model adjustment using *COOT* and refinement with *REFMAC* and *PHENIX*^40^ were performed in the last step to decrease the final *R*_work_/*R*_free_ factors to 0.21/0.27. The refined structure of ST50 was validated with *MolProbity*^41^. All residues are in the allowed region of the Ramachandran plot, except for Asp272 of the A protomer, which might result from the high flexibility of the extracellular loop.

## Additional Information

**Accession Codes**: The structural factors and coordinates have been deposited in the RCSB Protein Data Bank. The PDB code is 5BUN.

**How to cite this article**: Guan, H.-H. *et al.* Crystal structure of an antigenic outer-membrane protein from *Salmonella* Typhi suggests a potential antigenic loop and an efflux mechanism. *Sci. Rep.* **5**, 16441; doi: 10.1038/srep16441 (2015).

## Supplementary Material

Supplementary Information

## Figures and Tables

**Table 1:** Statistics of crystal diffraction and structure refinement.

**I. Crystal data**	ST50 (PDB entry 5 BUN)
Wavelength (Å)	0.9
Temperature (K)	110
Unit-cell parameters (Å)	*a = b = *246.85, *c* = 65.68
Resolution Range (Å) (outmost shell)	30–2.98 (3.03–2.98)^a^
Space group	*I*4
Unique reflections	40,826
Completeness (%)	99.8 (99.8)^a^
*I*/σ_*I*_	10.5 (1.1)^a^
Average redundancy	3.3
b*R*_*pim*_ (%)	7.5 (68.0)^a^
Mosaicity (^o^)	0.92
No. of protein molecules per A.U.	3
**II. Refinement results**	
c*R*_work_ (%)	20.96
d*R*_free_ (%)	27.51
Rmsd bond length (Å)	0.004
Rmsd bond angles (^o^)	0.931
Total number of residues (built)	467 (433)
Total number of protein atoms (non-hydrogen)	9971
Total number of β-OG	4
Ramachandran plot	
Most favored (%)	92.8
Allowed (%)	7.1
DisAllowed (%)	0.1

^a^The highest resolution shell.

^b^*R*_*pim*_ = Σ_hkl_([1/(n-1)]^1/2^) Σ_i_ [| I_i_ (hkl) -< I (hkl) > |/Σ_hkl_ Σ_i_ I_i_ (hkl)], where I_i_ is the *i*th measurement, <I(hkl)> is the weighted mean of all measurements of I (hkl) and n is the multiplicity of measurements.

^c^*R*_work_ = Σ_hkl_ | F_o_ − F_c_ |/Σ_hkl_ F_o_, where F_o_ and F_c_ are the observed and calculated structure factor amplitudes of reflections.

^d^*R*_free_, calculated the same as *R*_work_ but from a test set containing 5% of data excluded from the refinement calculation.
