# Supplementary material for: Crystal structure of an antigenic outer-membrane protein from Salmonella Typhi suggests a potential antigenic loop and an efflux mechanism
Source: Sci Rep. 2015 Nov 13;5:16441. doi: 10.1038/srep16441 (PMC4643347; doi:10.1038/srep16441)
Supplement: Supplementary Information [file srep16441-s1.pdf]

Supplementary information

Crystal structure of an antigenic outer-membrane protein from *Salmonella* Typhi suggests a potential antigenic loop and an efflux mechanism

Hong-Hsiang Guan[1], Masato Yoshimura[1], Phimonphan Chuankhayan[1], Chien-Chih Lin[1], Nai-Chi Chen[1,2], Ming-Chi Yang[1], Asma Ismail[3], Hoong-Kun Fun[4,5], and Chun-Jung Chen[1,2,4,6]*

Supplemental Figure 1

a

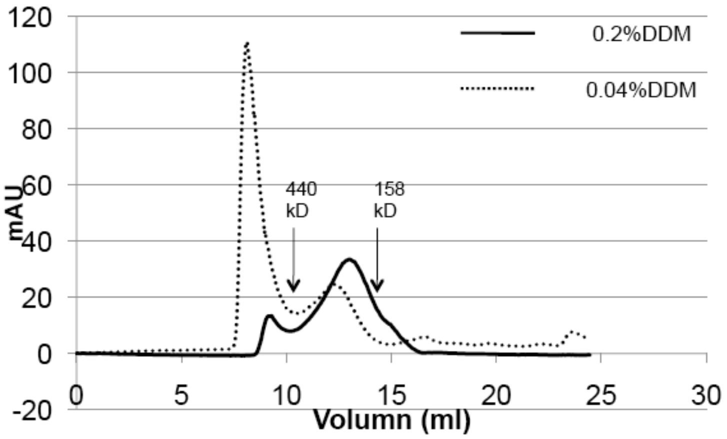

b

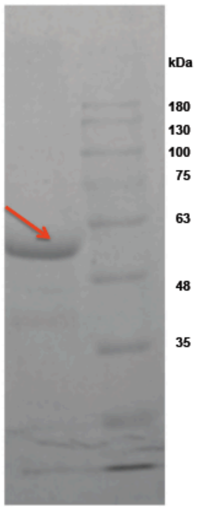

c

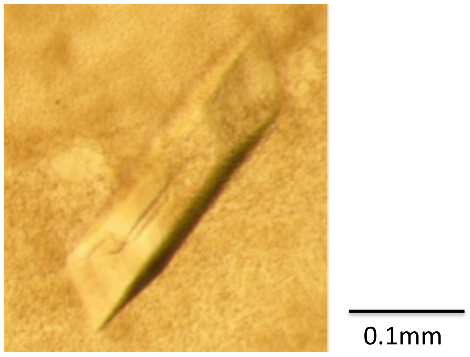

d

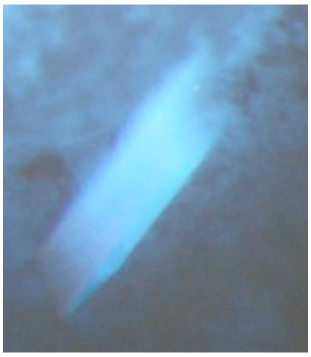

Purification and crystallization of ST50. (a) Size-exclusion chromatography of ST50 was conducted with the Superdex 200 in different concentrations (0.2 and 0.04%) of DDM. (b) The SDS-PAGE showed that the purity of ST50, labeled with the red arrow, was greater than 90%. The single crystal of ST50 was illuminated under the white light (c) and the UV light (d), respectively.

Supplemental Table 1

	Helix		Sheet		Coils	
**Prediction* Program**	No. of residues	Content (%)	No. of residues	Content (%)	No. of residues	Content (%)
**SAM**	339	72.6	47	10.0	81	17.4
**PSIPRED**	254	54.4	31	6.6	182	39.0
**PredictProtein**	270	57.8	27	5.8	170	36.4
**SABLE**	286	61.2	30	6.4	151	32.4
**YAPSIN**	272	58.3	41	8.8	154	32.9
**Average**	**284**	**60.8**	**36**	**7.7**	**147**	**31.5**

*The secondary structure prediction of ST50 (Δ24) with different prediction tools.

Supplemental Figure 2

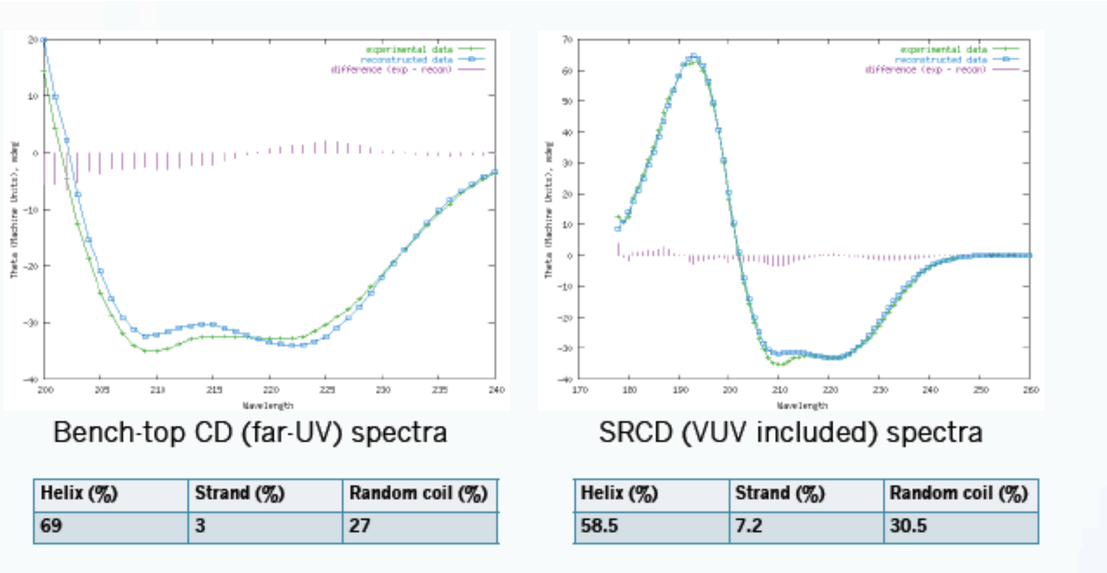

CD spectra of trimeric ST50. The conventional CD spectra (left panel) and SRCD spectra (right panel) were measured at the NSRRC_BL04 beamline and analyzed with algorithms *K2D* and *SELCON3*, respectively, at the *DichroWeb* website (http://dichroweb.cryst.bbk.ac.uk/). The green and blue lines represent the experimental spectra and calculated profile, respectively.

Supplemental Figure 3

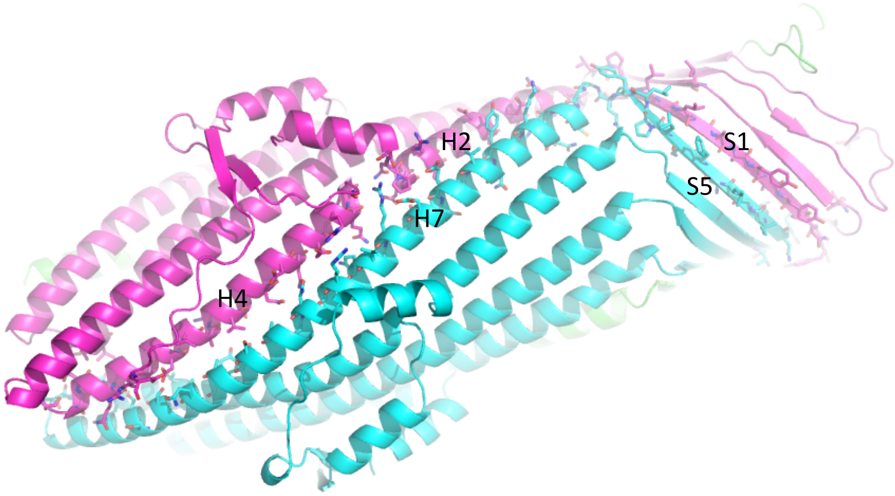

The intermolecular interface between two protomers. Two adjacent protomers of ST50 are colored pink and cyan, respectively. The interface residues between two adjacent protomers are shown in sticks.

Supplemental Figure 4

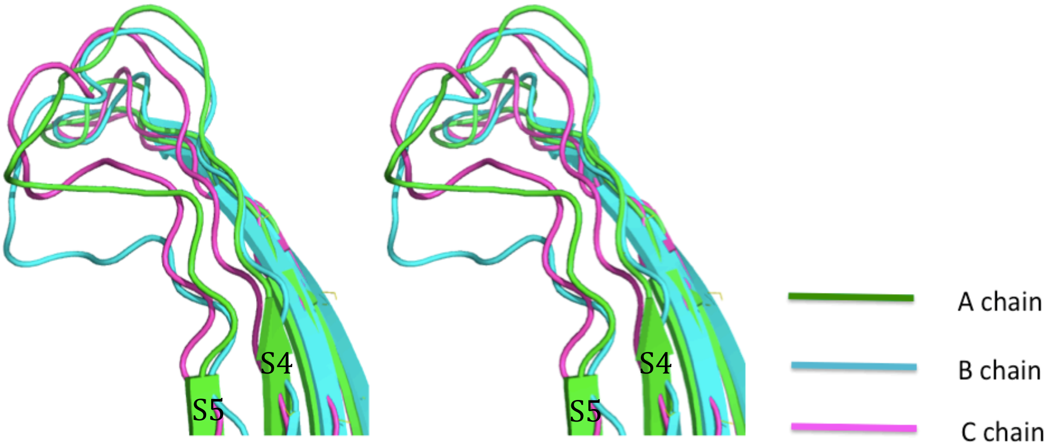

The stereo view of the superimposed extracellular loop 2 (L2) of each protomer of ST50. The protomers A, B and C of ST50 are colored as green, cyan, and pink respectively. The β-strands 4 and 5 are labeled as S4 and S5, respectively.

Supplemental Figure 5

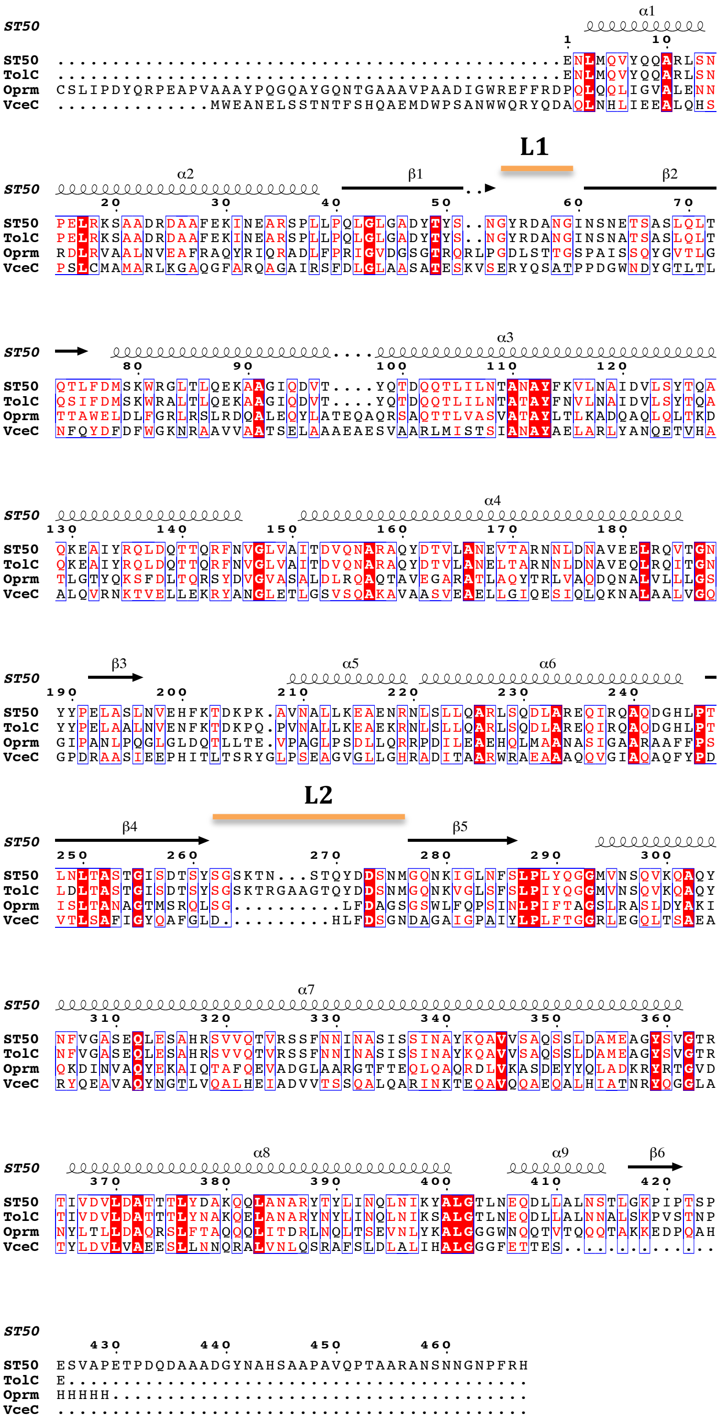

The sequence alignment of representative drug efflux outer-membrane proteins with known structures. The alignment was performed with *ClustalW2* (EMBL–EBI) (http://www.ebi.ac.uk/Tools/msa/clustalw2/) and *ESPript* (http://espript.ibcp.fr/). α denotes the α-helix and β denotes the β-strand. The orange lines represent the extracellular loop 1 (L1) and the extracellular loop 2 (L2), respectively.

Supplemental Figure 6

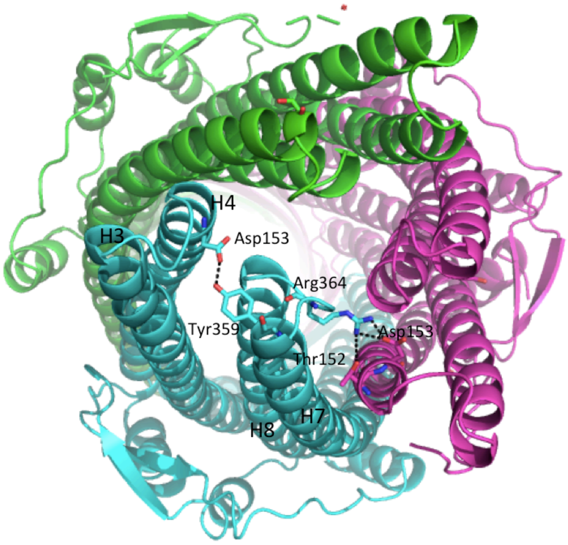

The hydrogen-bond network of the periplasmid entrance in ST50 in the resting closed state. The hydrogen-bond network in ST50 is composed of four key residues – Thr152, Asp153, Tyr359 and Arg364. The dotted lines denote hydrogen bonds. Helices 3, 4, 7 and 8 are labeled as H3, H4, H7 and H8, respectively.

Supplemental Figure 7

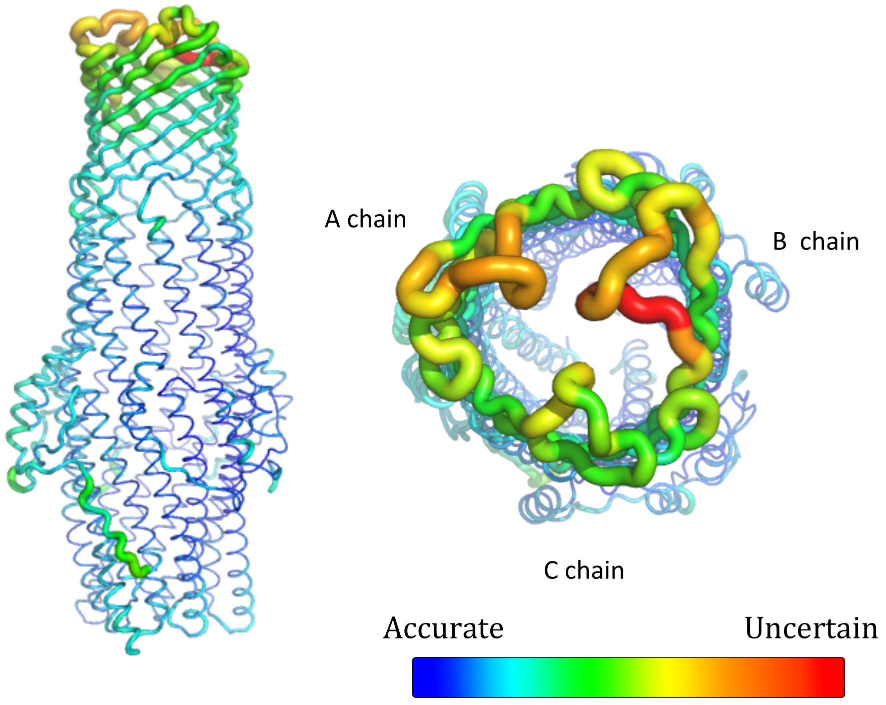

***B*-factor putty presentation of ST50**. The color gradient represents the temperature *B*-factor variation from the small values (blue color, accurate or rigid structure) to the large values (red color, uncertain or flexible structure).
